# Mitochondrial calcium uniporter protein MCU is involved in oxidative stress-induced cell death

**DOI:** 10.1007/s13238-015-0144-6

**Published:** 2015-03-11

**Authors:** Yajin Liao, Yumin Hao, Hong Chen, Qing He, Zengqiang Yuan, Jinbo Cheng

**Affiliations:** 1 State Key Laboratory of Brain and Cognitive Sciences, Institute of Biophysics, Chinese Academy of Sciences, Beijing, 100101 China; 2 College of Life Sciences, University of Chinese Academy of Sciences, Beijing, 100049 China

**Keywords:** MCU, VDAC1, oxidative stress, calcium uptake, cell death

## Abstract

Mitochondrial calcium uniporter (MCU) is a conserved Ca^2+^ transporter at mitochondrial in eukaryotic cells. However, the role of MCU protein in oxidative stress-induced cell death remains unclear. Here, we showed that ectopically expressed MCU is mitochondrial localized in both HeLa and primary cerebellar granule neurons (CGNs). Knockdown of endogenous MCU decreases mitochondrial Ca^2+^ uptake following histamine stimulation and attenuates cell death induced by oxidative stress in both HeLa cells and CGNs. We also found MCU interacts with VDAC1 and mediates VDAC1 overexpression-induced cell death in CGNs. This finding demonstrates that MCU-VDAC1 complex regulates mitochondrial Ca^2+^ uptake and oxidative stress-induced apoptosis, which might represent therapeutic targets for oxidative stress related diseases.

## INTRODUCTION

As the major source of ATP, mitochondrion plays an essential role in cellular physiology and metabolism in eukaryotic cells. On the one hand, low mitochondrial Ca^2+^ concentration fails to effectively activate pyruvate dehydrogenase as to produce enough ATP (Robb-Gaspers et al., 1998); on the other hand, overloaded mitochondrial Ca^2+^ reduces mitochondrial membrane potential (Δψ) and triggers cell death. Therefore, mitochondrial Ca^2+^ homeostasis is essential for physiological function and survival of the cells.

Some proteins involved in mitochondrial Ca^2+^ uptake have been discovered in recent years. VDAC (voltage-dependent anion channel) is the first identified Ca^2+^ transport channel, which locates in the outer membrane of mitochondria (OMM) and highly permeable to Ca^2+^ (Xu et al., 1999). VDAC1 will lose its Ca^2+^ transport capacity when the Ca^2+^ binding sites are blocked by Ruthenium Red (RuR) (Gincel et al., 2001). MCU is another identified Ca^2+^ uniporter protein, which is localized in the inner membrane of mitochondrion (IMM) (Baughman et al., 2011). Numbers of proteins have been shown to regulate the activity of MCU, such as essential mitochondrial calcium uptake1/2 (MICU1/2), MCU regulator (EMRE), MCUb and MCU regulator 1 (MCUR1) (Ahuja and Muallem, 2014; Alam et al., 2012; Mallilankaraman et al., 2012a; Mallilankaraman et al., 2012b; Raffaello et al., 2013; Sancak et al., 2013). When cells undergo severe damage, mitochondrial permeability transition pore (PTP) will open and release pro-apoptotic factors, especially cytochrome c, from mitochondria to the cytoplasm (Kinnally et al., 2011). Overexpression of VDAC1 renders cells sensitive to oxidative stress inducers, such as thapsigargin (TG) and arsenic trioxide (As2O3) (Ben-Hail and Shoshan-Barmatz, 2012; Shoshan-Barmatz et al., 2010; Shoshan-Barmatz et al., 2009). Ectopically expressed VDAC1 increases the concentration of Ca^2+^ in the inner-membrane space of mitochondrion, leading to mitochondrial Ca^2+^ overload and cytochrome c release (Brustovetsky et al., 2002; Madesh and Hajnoczky, 2001; Naranmandura et al., 2012; Rapizzi et al., 2002). Cytochrome c interacts with Apaf1 and initiates activation of caspase-3 (Chu et al., 2001). As a subunit of the PTP, VDAC1 is involved in cytochrome c release and apoptosis (Zheng et al., 2004). Whether MCU, the inner membrane Ca^2+^ uniporter, has the similar function as VDAC1 protein in neurons is poorly studied. In our study, we found that MCU plays an important role in oxidative stress-induced apoptosis. In addition, we showed that MCU interacts with VDAC1 and is involved in the VDAC1-mediated cell death in CGNs.

## RESULTS

### MCU regulates the mitochondrial Ca^2+^ uptake in primary CGNs

**Figure 1:**
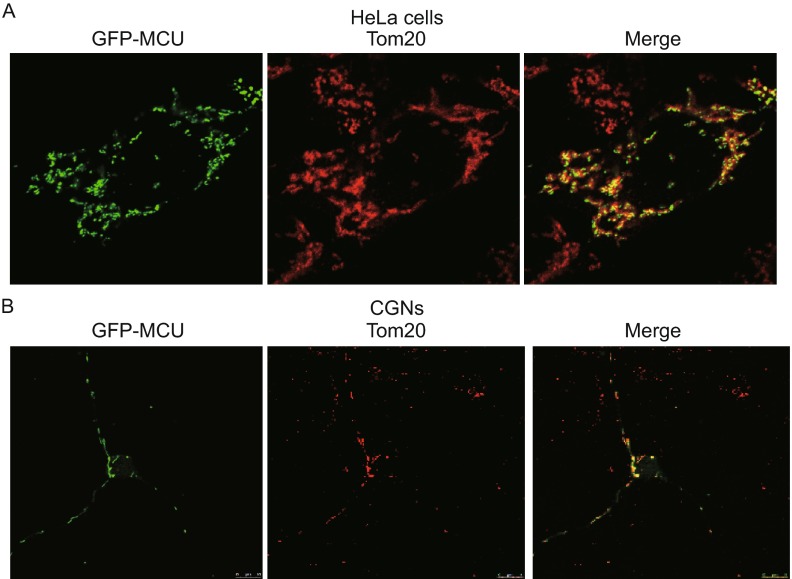
**The sublocation of MCU in HeLa cells and CGN**. (A) GFP-MCU (green) was tranfected into HeLa cells. Twenty-four hours post transfection, cells were fixed and stained with Tom20 (Red). (B) GFP-MCU (green) and mito-Red (red) were co-tranfected into CGNs. Twenty-four hours post transfection, cells were fixed and imaged by laser confocal microscope using a 40× objective lens

**Figure 2:**
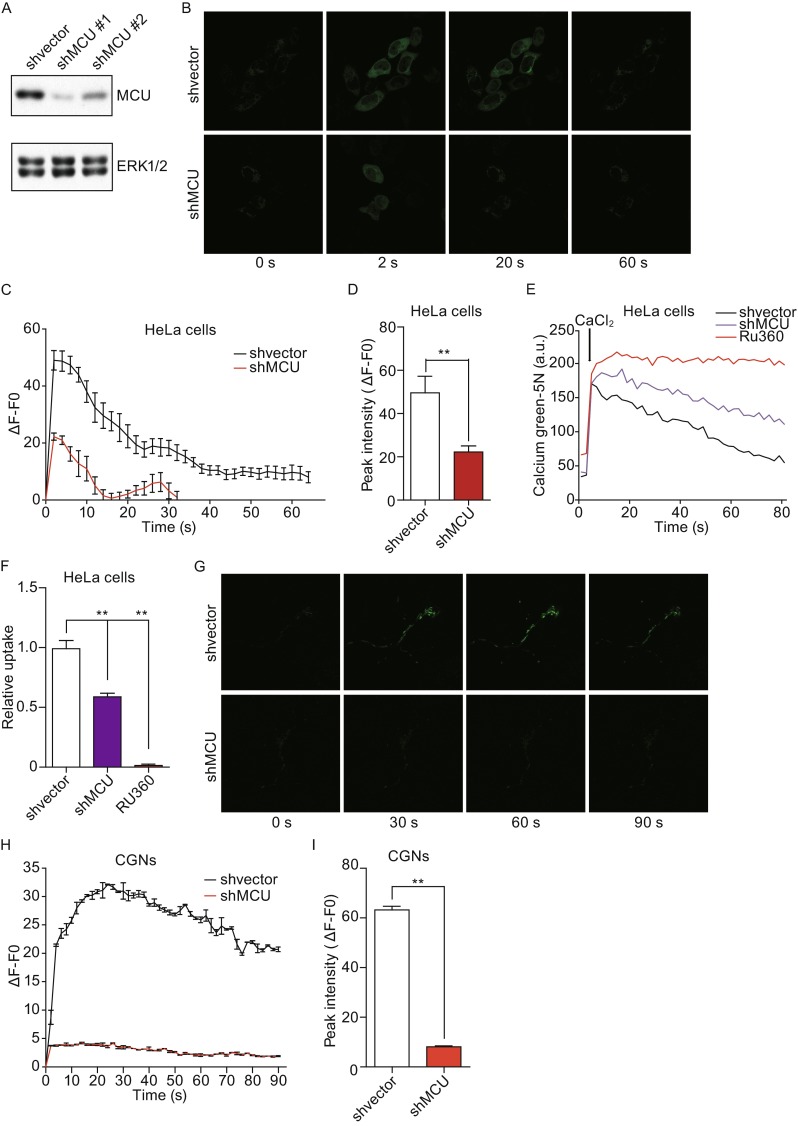
**MCU is required for mitochondrial calcium uniporter in HeLa cells and CGNs**. (A) Two shRNA against MCU were designed to knockdown MCU in HeLa cells and the knockdown efficiency of each was detected by Western blot. (B) Knocking down MCU decreased mitochondrial Ca^2+^ uptake by assaying Mito-GCaMP3. Control or MCU knockdown HeLa cells were transfected with Mito-GCaMP3. GCaMP3 fluorescence was measured before and during exposure to histamine stimulation (100 μmol/L). (C) Representative traces Ca^2+^ response to histamine stimulation in control or MCU knockdown HeLa cells. (D) Graph shows quantification of peak Ca^2+^ intensities. (E) Representative traces of Ca^2+^ uptake in digitonin-permeabilized HeLa cells. The level of Ca^2+^ was indicated by the calcium Green-5N. (F) Graph shows relative Ca^2+^ uptake. (G) CGNs were transfected with shMCU or shvector together with Mito-GCaMP3. GCaMP3 fluorescence was measured before and after exposure to histamine (100 μmol/L). (H) Representative traces showing Ca^2+^ response after histamine stimulation over time in MCU wild type or knockdown cells were. (I) Graph shows quantification of peak Ca^2+^ intensities

First, we found that overexpressed MCU was mainly localized in the mitochondria in HeLa cells and CGNs (Fig. 1A and 1B). To identify whether MCU functions as mitochondrial Ca^2+^ uniporter in HeLa cells, two shRNAs against MCU were designed. As shown in Fig. 2A, shRNA#1 had a better knockdown efficiency compared with shRNA#2. In order to monitor mitochondrial Ca^2+^ levels, we transfected cells with a mitochondrial targeted Ca^2+^ indicator, Mito-GCaMP3. Knockdown of MCU dramatically decreased histamine-induced mitochondrial Ca^2+^ level, which includes both the peak Ca^2+^ level and the recovery time to baseline (Fig. 2B–D). Furthermore, the effect of MCU knockdown on mitochondrial Ca^2+^ uptake was examined in digitonin-permeabilized cells. In normal condition, mitochondria could rapidly clear most of externally added 50 µmol/L Ca^2+^. However, MCU knockdown remarkably inhibited external Ca^2+^ clearance, similar to Ru360, a specific MCU blocker (Fig. 2E and 2F). Consistently, in CGN cells transfected with Mito-GCaMP3, MCU knockdown dramatically blocked mitochondrial Ca^2+^ uptake (Fig. 2G–I). Together, these findings implicate MCU functions as a mitochondrial Ca^2+^ uniporter in both HeLa cells and CGNs.

### MCU is involved in oxidative stress-induced cell death

**Figure 3:**
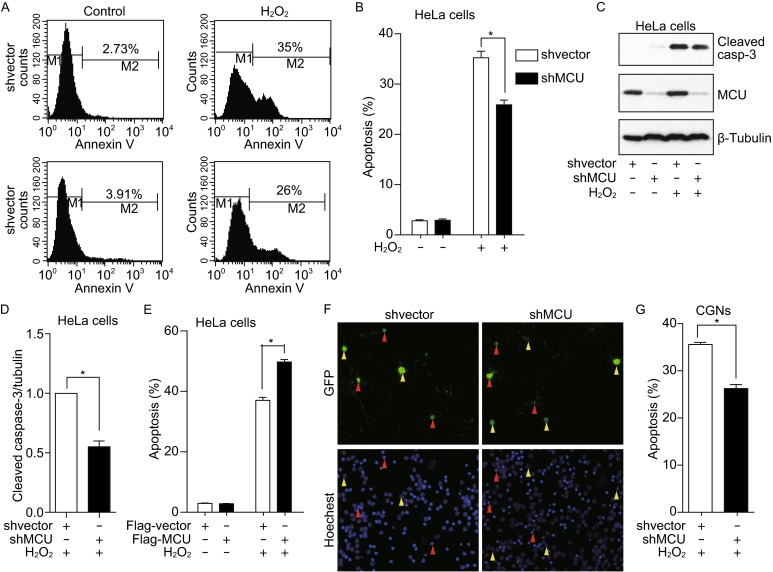
**MCU is involved in oxidative stress-induced cell death**. (A and B) MCU silenced HeLa cells or control cells were treated with H_2_O_2_ for 24 h, then, cells were collected and stained with FITC labeled annexin-V and finally analysis by flow cytometer. (C) MCU silenced HeLa cells or control cells were treated with H_2_O_2_ for 24 h, and cells were collected and the cleaved caspase-3 was detected. (D) Graph shows normalized level of cleaved caspase-3. (E) HeLa cells tranfected with Flag-vector or Flag-MCU were exposed to H_2_O_2_ for 24 h and apoptosis was analyzed by FACS. (F and G) CGNs transfected with GFP vector together with shMCU or shvector were treated with 70 μmol/L H_2_O_2_ for 24 h. Green arrowhead stands for the healthy cells and red arrowhead indicates apoptotic cells

Next, we studied the role of MCU in oxidative induced-cell death. As shown in Fig. 3A and 3B, MCU knockdown decreased hydrogen peroxide (H_2_O_2_)-induced apoptosis. Caspase-3 activation was also reduced in MCU knockdown cells (Fig. 3C and 3D). Accordingly, MCU overexpression significantly increased apoptosis in HeLa cells treated with H_2_O_2_ (Fig. 3E). We also found that MCU knockdown decreased the H_2_O_2_-induced cell death in CGNs as shown in Fig. 3F and 3G. Taken together, these results suggested that MCU is involved in oxidative stress-induced cell death.

### MCU Ca^2+^ uptake activity is required for oxidative stress-induced cell death

**Figure 4:**
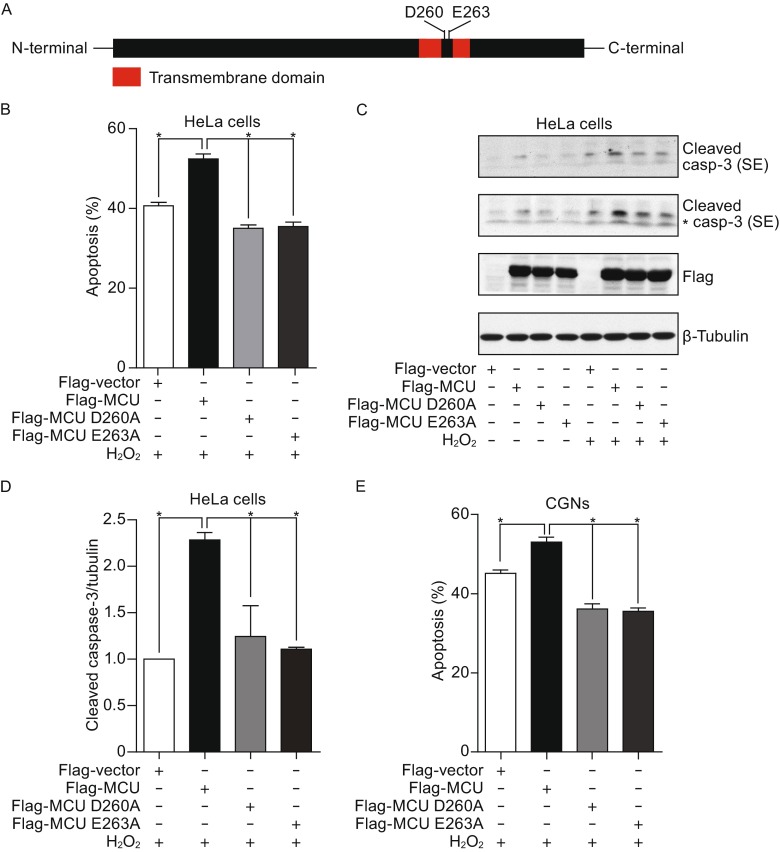
**The calcium uptake activity of MCU is required for oxidative stress-induced cell death**. (A) The critical site for calcium uptake activity of MCU. (B) HeLa cells were overexpressed with wild type MCU, MCU_D260A_ or MCU_E263A_, and then cells were treated with H_2_O_2_ for 24 h. The apoptosis was finally analyzed by FACS. (C) HeLa cells transfected with wild type MCU, MCU_D260A_ or MCU_E263A_ as indicated were treated with 200 μmol/L H_2_O_2_ for 24 h. Cleaved caspase-3 were analyzed (“*” means non-specific band). (D) Graph shows normalized level of cleaved caspase-3. (E) CGNs transfected with GFP vector plus wild type MCU, MCU_D260A_ mutant, MCU_E263A_ mutant or empty vector were treated with 70 μmol/L H_2_O_2_ for 24 h

It has been reported that D260 and E263 of MCU are critical for the regulation of Ca^2+^ uptake (Fig. 4A) and the mutations of MCU_D260A_ and MCU_E263A_ lose the Ca^2+^ uptake activity (Baughman et al., 2011). To further confirm whether the function of Ca^2+^ uptake activity of MCU was required for oxidative stress-induced cell death, we constructed the MCU mutants and transfected into cells. We observed that overexpression of wild type MCU significantly increased cell death induced by H_2_O_2_. However, expression of MCU_D260A_ mutant or MCU_E263A_ mutant failed to exaggerate oxidative stress-induced apoptosis (Fig. 4B). In addition, we found that overexpression of wild type MCU, not the mutants, increased caspase-3 cleavage (Fig. 4C and 4D). Consistently, we also found that MCU overexpression increased cell death induced by H_2_O_2_ in primary cultured CGNs, and the mutants of MCU_D260A_ and MCU_E263A_ had no effect on the apoptosis (Fig. 4E). Thus, these observations indicated that the Ca^2+^ uptake activity of MCU is required for oxidative stress-induced cell death.

### MCU interacts with VDAC1 and functions as a downstream of VDAC1 during oxidative stress-induced cell death

**Figure 5:**
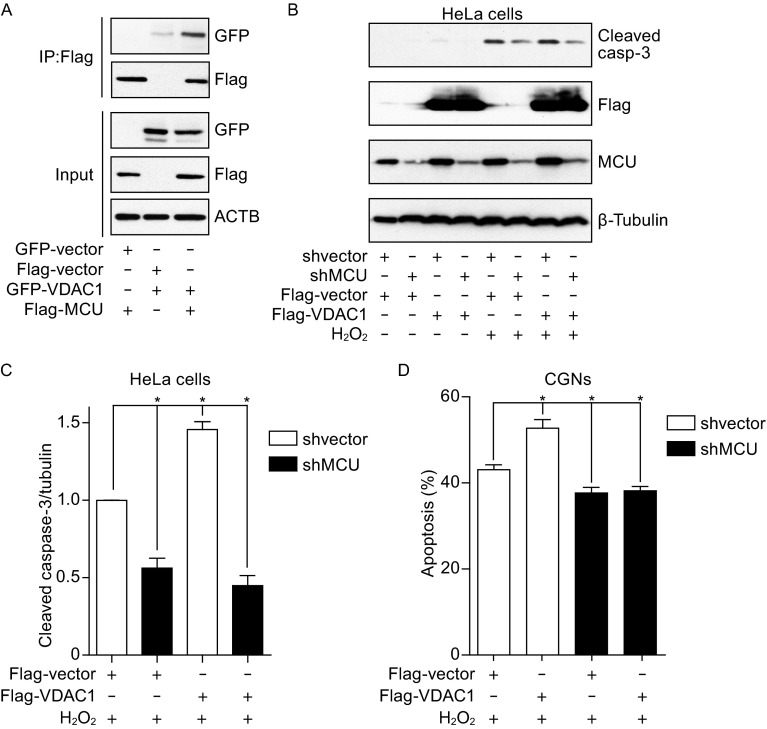
**MCU interacts with VDAC1 and functions as a downstream of VDAC1**. (A) Flag-MCU was transfected into cells together with GFP-VDAC1 or empty vector. Twenty-four hours later, cells were collected and immunoprecipitated using anti-Flag M2 beads. (B) Normal or MCU knockdown HeLa cells were transfected with Flag-VDAC1 or empty vector. Twenty-four hours later, cells were treated with 200 μmol/L H_2_O_2_ for another 24 h and cleaved caspase-3 was analyzed. (C) Graph shows normalized level of cleaved caspase-3. (D) CGNs transfected with shMCU or shvector, Flag-VDAC1 or empty vector as indicated were treated with 70 μmol/L H_2_O_2_ for 24 h

It has been shown that VDAC1 overexpression made cells sensitive to oxidative stress (Rapizzi et al., 2002). Since both VDAC1 and MCU are involved in mitochondrial calcium uptake, we then ask whether there is functional interaction between two proteins. We firstly observed there is a physical interaction of MCU and VDAC1 (Fig. 5A). Next, we investigated the biological function of MCU-VDAC1 interaction in cells. We found that overexpression of VDAC1 increased the apoptosis induced by H_2_O_2_ in HeLa cells and MCU knockdown significantly inhibited VDAC1 overexpression-induced cell death, which suggests MCU functions as the downstream of VDAC1 during oxidative stress-induced apoptosis (Fig. 5B and 5C). VDAC1 has also been shown to be involved in neuronal cell death (Fernandez-Echevarria et al., 2014). Here, we found that overexpression of VDAC1 increased oxidative stress-induced apoptosis in CGNs and knockdown of MCU significantly mitigated VDAC1 overexpression-induced cell death (Fig. 5D). Taken together, these results suggested MCU functions as the downstream of VDAC1 during oxidative stress-induced cell death.

## DISCUSSION

In this study, we demonstrate the role of MCU in oxidative stress-induced cell death by loss- and gain-of-function experiments. We found MCU interacts with VDAC1 and functions as the downstream in the process of oxidative stress-induced cell death.

Oxidative stress is contributed to the pathogenesis of neurological diseases, such as stroke and degenerative diseases. Mitochondrial calcium overload plays an important role in the oxidative stress-induced neuronal death. In our study, we found MCU is localized at mitochondria and functioned as a crucially Ca^2+^ channel in both HeLa and primary CGNs. MCU knockdown significantly blocked the mitochondrial Ca^2+^ uptake activity. Moreover, our functional studies indicate MCU is involved in oxidative stress-induced cell death. Recently, Pan et al. generated MCU^-/-^ mice and found that MCU^-/-^ mice are grossly normal (Pan et al., 2013), but a significant reduction of mitochondrial matrix calcium. In this study, we found that MCU knockdown did not affect the cell growth in both HeLa and primary CGNs, but rendered the cells resistant to oxidative stress. Mitochondrial Ca^2+^ overload is usually observed during ischemia/reperfusion (I/R), and it is considered to aggravate I/R injury. As MCU is a mitochondrial Ca^2+^ uniporter, inhibition of MCU activity might be a therapy strategy for oxidative stress-induced diseases. For example, inhibition of MCU activity has been demonstrated to attenuate I/R injury in multiple organs including brain, heart and liver (Dong et al., 2014; Schwartz et al., 2013; Zhao et al., 2013). Inhibition of MCU activity also protects brain and heart from iron overload-induced dysfunction (Kumfu et al., 2012; Sripetchwandee et al., 2013a; Sripetchwandee et al., 2014; Sripetchwandee et al., 2013b). Accordingly, enhancement of MCU activity increases Ca^2+^ level in mitochondria and promotes oxidative stress-induced apoptosis (De Stefani et al., 2011). Interestingly, recent studies showed that the role of MCU in apoptosis is dependent on cell type. Knockdown of MCU protect HeLa cells, not in MEF cells, against oxidative stress induced apoptosis (De Stefani et al., 2011; Hall et al., 2014; Pan et al., 2013). In this study, we found that MCU knockdown attenuates oxidative stress-induced apoptosis in both HeLa and primary CGNs.

PTP is regarded as the gatekeeper of apoptosis, and its opening is regulated by several proteins, such as VDAC1, adenosine nucleotide translocase (ANT) and cyclophilin D (Halestrap et al., 1997; Pestana et al., 2010). Overexpression of these proteins makes cells sensitive to apoptotic-inducing stimulus. Overexpressed VDAC1 in HeLa cells enhances cell death upon treatment with H_2_O_2_, staurosporine (STS), TG or As_2_O_3_ (Keinan et al., 2013). In this study, we found MCU knockdown remarkably inhibited VDAC1 overexpression induced-cell death, suggesting MCU functions as a downstream of VDAC1 during oxidative stress-induced cell death.

In summary, we showed that VDAC1 (outer member) and MCU (inner member) form complex and mediate mitochondrial calcium uptake and stress-induced cell death. Furthermore, we showed that MCU is involved in oxidative stress-induced apoptosis as a downstream regulator of VDAC1. Therefore, the inhibition of the activity of MCU or disruption of VDAC1-MCU interaction might be a strategy to stroke and degenerative diseases.

## MATERIALS AND METHODS

### Materials

H_2_O_2_, anti-Flag M2 mAb and Anti-MCU pAb were purchased from Sigma-Aldrich. Anti-active caspase-3 pAb was purchased from Millipore. Anti-GFP pAb, anti-GAPDH mAb, anti-β-tubulin mAb were purchased from CWbiotech. Primers for shRNA were synthesized by Invitrogen and cloned into pLKO.1 vector.

### Cell culture and transfection

HeLa cells were maintained in DMEM supplied with 10% fetal bovine serum at 37°C in a humidified atmosphere with 5% CO_2_. Mouse primary cerebellar granule neurons (CGNs) were isolated from 10 days old mouse as previously described (Xie et al., 2012). Isolated CGNs were cultured in BME supplemented with 10 μmol/L cytosine arabinoside (AraC) and 25 mmol/L glucose. Lipofectamine 2000 reagent was applied for transfection in HeLa cells according to the manufacturer’s instructions. For transfection of CGNs, the classical calcium phosphate coprecipitation technique was used.

### Imaging of mitochondrial calcium

Mitochondrial Ca^2+^ uptake in intact cells was detected as previously described (Qiu et al., 2013). Briefly, cells plated on 35-mm glass-bottom dishes were transfected with Mito-GCaMP3 to monitor the concentration of Ca^2+^ in mitochondrial. 24 h post transfection, time-lapse confocal microscopy was started at 1-sec intervals using a 40× objective lens. Images were obtained using laser scanning confocal microscope. GCaMP3 was excited using the 488 nm line of an argon laser and detected at 530–550 nm. Ten to fifteen cells were randomly selected in each scan by drawing regions around individual cells, and the green fluorescence intensity was monitored sequentially. The results are representative of at least three independent experiments, and we have confirmed the reproducibility of these findings.

### Calcium uptake in permeabilized HeLa cells

Mitochondrial Ca^2+^ uptake in permeabilized cells was tested as previously described (Sancak et al., 2013). Briefly, HeLa cells grown in 10 cm tissue culture plates were trypsinized and resuspended in 10 mL of DMEM. One million of each cell lines were transferred to microcentrifuge tubes, followed by spining down for 3 min at 800 ×*g* at room temperature. Then cells were washed with PBS once and resuspended in KCl buffer (125 mmol/L KCl, 2 mmol/L K_2_HPO_4_, 1 mmol/L MgCl_2_, 20 mmol/L HEPES, pH 7.2), supplemented with 5 mmol/L glutamate and malate, 0.01% digitonin and 0.8 μmol/L Green-5N. Fluorescence was monitored every 0.2 s before and after addition of 50 μmol/L final concentration of Ca^2+^ at 27°C using a Thermo Scientific Varioskan Flash, filter sets (Ex506/Em532).

### Immunoblot analysis

Cells were lysed in RIPA buffer (strong) (biyuntian, China). The concentration of total proteins was determined using BCA protein concentration detection kit (biyuntian, China). The same amount of proteins were loaded on SDS-PAGE and resolved by electrophoresis, followed by transferring onto NC membrane. Then, the membranes were blocked with blocking buffer (5% fat-free dry milk in TBST buffer) and incubated with primary antibodies and HRP labeled secondary antibodies, respectively. Lastly, specific proteins were visualized with enhanced ECL plus Western blotting substrate according the manufacturer’s instructions (Thermo).

### Apoptosis analysis

For HeLa cells, apoptotic cells were quantified by flow cytometry using an Annexin-V-FITC apoptosis detection kit (biyuntian) following the manufacturer’s instructions. For CGNs, apoptotic cells were detected by nuclei staining using Hoechest 33342. Primary CGNs were isolated and seeded on glass piece. Seven days later, cells were transfected with plasmid or shRNA. Forty-eight hours later, cells were treated with 70 μmol/L H_2_O_2_ for 24 h, and then cells were fixed and stained with Hoechest 33342. Neuronal apoptosis assay was performed as described (Konishi and Bonni, 2003) by using the Zeiss imager D1 microscope.

### Statistical analysis of data

Statistical data are presented as mean ± S.D. Significance was calculated by Student’s *t*-test (* means *P* < 0.05, ** means *P* < 0.01).
